# Failure to return to receive HIV-test results: the Cameroon experience

**DOI:** 10.1186/s13104-017-2632-7

**Published:** 2017-07-24

**Authors:** Defo Defo, Emmanuel Armand Kouotou, Jobert Richie Nansseu

**Affiliations:** 1 Yaoundé Central Hospital, Yaoundé, Cameroon; 2 Department of Internal Medicine and Specialties, Faculty of Medicine and Biomedical Sciences, University of Yaoundé I, Yaoundé, Cameroon; 3 Yaoundé University Teaching Hospital, Yaoundé, Cameroon; 4 Sickle Cell Disease Unit, Mother and Child Centre of the Chantal Biya Foundation, Yaoundé, Cameroon; 5 Department of Disease, Epidemics and Pandemics Control, Ministry of Public Health, Yaoundé, Cameroon; 6 Department of Public Health, Faculty of Medicine and Biomedical Sciences, University of Yaoundé I, PO Box 1364, Yaoundé, Cameroon

**Keywords:** Failure to retrieve, HIV-test result, Cameroon, Sub-Saharan Africa

## Abstract

**Background:**

To be acquainted of one’s HIV serological status is an important strategy for the fight against HIV/AIDS, but many patients still miss the appointment to receive their HIV-test result. The aim of this study was to determine the epidemiological and serological profiles of patients not coming back to collect their HIV-test result in a tertiary health care facility in Yaoundé, Cameroon.

**Methods:**

We undertook a retrospective analysis of data of patients who visited the HIV-day care unit of the Yaoundé Central Hospital (Yaoundé, Cameroon) from January to December 2009 in order to be screened for HIV, and who did not come back to retrieve their result.

**Results:**

Of the 3990 patients who visited the unit during the study period, 396 (9.92%) did not come back to collect their HIV-test result, of whom 21% were aged 26–30 years representing the most encountered age group. Females (57.8%) were significantly more present than males (p = 0.023). More than half of these patients (54.9%) visited the unit for a voluntary HIV testing. Two hundred and three patients (51.3%) had a positive HIV-test result, of whom 63.5% were females. The most HIV-infected group was the one coming for a voluntary screening (36.5%). Women not returning to receive their results were more likely to be tested HIV-positive (odds ratio = 1.62, 95% confidence interval: 1.08–2.42; p < 0.05).

**Conclusions:**

Almost 10% of patients screened for HIV infection did not come back to collect their result. More strategies should be implemented to reduce this rate, especially targeting young adults (20–35 years) and women. The delay in delivering patients’ results should be shortened. Concurrently, there is need to increase voluntary HIV-testing in our settings.

## Background

To date, the HIV/AIDS pandemic remains a scourge and pending problem in Africa, especially in the sub-Saharan part of the continent. Fortunately, the World Health Organization (WHO) reports reveal that the incidence of HIV infection keeps on decreasing, though the trends may vary from one region to another [1, 2]. The advent and widespread use of highly active antiretroviral therapy (HAART) in clinical practice has indeed considerably modified the natural history of HIV infection by significantly improving the prognosis and quality of life of people living with the disease, with a consequential drastic reduction in the morbidity and mortality related to HIV and its complications [1, 2].

Being acquainted of one’s HIV serological status is one of the foremost strategies put in place for the fight against HIV/AIDS as it can lead to the change in HIV-risky behaviors, hence a significant reduction in the disease spreading [3]. This involves a voluntary screening for the detection of the infection along with a counselling targeting all aspects of the disease. Knowing one’s HIV serological status is very important in order to get access to the right management including an adequate treatment and a tight follow-up, and to be prevented from any further contamination or subsequent transmission of the infection. In fact, there is now strong evidence claiming that patients on efficient antiretroviral therapy medication limit the transmission of HIV infection [4].

Regrettably, a large number of voluntary testing campaigns do not reach all those at risk. Further, when these persons at risk of HIV-infection have accepted to be tested, many of them will never come back to collect their result, mainly due to the fear of stigmatization if the result appears positive [5, 6]. In resource-limited settings, it has been observed for instance that one of the reasons restraining patients from coming back to retrieve their result is the lack of money to take a means of transport. It has been suggested, to overcome this difficulty, to ring patients and communicate them their result by telephone [7]. Although this solution may appear feasible and efficient, it does not permit to manage a potential shock that could accompany the announcement of such news to a patient, and it supposes by the way that all people possess a telephone by which they could be reachable, something that is untrue especially in remote and/or resource-poor areas.

Reasons explaining failure to retrieve HIV-test results have not been extensively investigated in Cameroon, a sub-Saharan African country. The present study was thus undertaken, seeking at determining the epidemiological and serological profiles of patients not returning to receive their HIV-test result.

## Methods

### Study design, setting and participants

This retrospective cross-sectional survey was conducted at the HIV-day care unit of the Yaoundé Central Hospital, Cameroon, from January to December 2009. This is a specialised clinic, located inside the Yaoundé Central Hospital, and dedicated to the prevention and management of HIV infection. The large majority of HIV-infected patients residing in or around Yaoundé are followed-up in this clinic, around 40,000 people to date. Data were recorded from standardized data collection forms of patients who visited the clinic in order to be screened for HIV infection during the study period, but who never came back to collect their result.

A standardized data collection form is available at the study site for any patient coming to be screened for HIV infection. This form records socio-demographic data (age, sex, occupation, marital status, residence), reasons for screening (voluntary, travelling, confirmation of a positive test, prenuptial assessment, or any associated disease [i.e. justifying the screening like tuberculosis or zona] among others), and information on pre- and post-test counselling. Prior to HIV-testing, each patient must undergo a pre-test counselling, first collective and then individual, the aim of which is to evaluate the patient’s knowledge on HIV infection. Additionally, the patient is questioned on the possible different strategies that could be put in place to cope with the newly discovered status, and the possibility to receive a close medical and psycho-social follow-up if the patient is tested positive.

At the end of the pre-test counselling, a clear approval is obtained from each patient before performing any blood sampling to screen for HIV-1 and HIV-2 antibodies. Only rapid diagnostic tests are used, the first of which has a sensitivity of almost 100%. When this first test is positive, a second one, which has a specificity approaching 100% and uses a different principle, is performed. It is only when these two tests are positive to HIV antibodies that the patient is declared HIV infected. After blood collection, an appointment is given to the patient within 3 days, for him/her to come back and get his/her result, something which cannot be done without undergoing a post-test counselling. The pre- and post-test counsellings are undertaken by trained psycho-social counsellors. The 3-day delay in giving back the patient’s result was explained by two main reasons: (1) the heavy workload: the clinic is highly solicited and the personnel has to receive patients coming to be tested and those already known infected who come for their regular follow-up visits; (2) the insufficient number of (trained) personnel working at the clinic: psycho-social counsellors (who had to conduct the pre-test and more importantly the post-test counselling); laboratory technicians (who had to sample the patients and perform the test), biologists (who confirmed, validated and signed patients’ results), and medical secretaries (who had to computerize patients’ records including their result).

### Ethical considerations

We obtained approvals from the Ethical Review Board of the Yaoundé Central Hospital and the Institutional Ethical Review Board of the Faculty of Medicine and Biomedical Sciences of the University of Yaoundé I, Cameroon. As the study was retrospective; we could not obtain any participant’s consent. Nonetheless, the need for patients’ consent was waived by both ethical review boards. The present manuscript was written in accordance with the Strengthening the Reporting of Observational Studies in Epidemiology (STROBE) guidelines [8]. Confidentiality of patients’ results was preserved.

### Statistical methods

Data analyses used Epi Info version 3.5.3 (CDC, Atlanta, USA). Quantitative variables are expressed as means and standard deviation (SD) whereas qualitative ones, as counts and percentages. Variables comparison used the Student’s t test or non-parametric equivalents for quantitative variables, and the Chi square test and equivalents for qualitative variables where appropriate. Odds ratios (OR) with 95% confidence intervals (CI) were used to investigate the impact of various factors on the refusal to withdraw HIV test results. A p < 0.05 served to characterize statistically significant results.

## Results

Of the 3990 patients screened for HIV infection during the study period, 396 (9.9%) did not come back to collect their result. Their ages ranged from 15 to 76 years with a mean of 32 (2.1) years old. Females were significantly more represented than males (57.8% vs. 42.2%, p = 0.023). People aged 26–30 years (21%) were those who did not come back to receive their results the most. Table 1 depicts the different reasons motivating the willingness of patients to be tested for HIV: voluntary testing (53.5%) and associated diseases (21.5%) were the prevailing ones.

**Table 1:** Reasons for HIV-testing matched with HIV-test results

Reasons	HIV-test result		Total N (%)
	Negative N (%)	Positive N (%)	
Accident of exposure to blood	4 (2.1)	2 (1.0)	6 (1.5)
Before surgery	1 (0.5)	0 (0.0)	1 (0.3)
Reminiscent disease	17 (8.8)	68 (33.5)	85 (21.5)
Confirmation	3 (1.6)	34 (16.7)	37 (9.3)
Control	3 (1.6)	5 (2.5)	8 (2.0)
During pregnancy	6 (3.1)	1 (0.5)	7 (1.8)
Prenuptial assessment	8 (4.1)	9 (4.4)	17 (4.3)
Risky-sexual contact	1 (0.5)	6 (3.0)	7 (1.8)
Voluntary	138 (71.5)	74 (36.5)	212 (53.5)
Traveling	3 (1.6)	0 (0.0)	3 (0.8)
Other (not mentioned)	9 (4.7)	4 (2.0)	13 (3.3)

We observed among the 3990 patients visiting the clinic a HIV seroprevalence of 43.8%. Specifically, 203 (51.3%) patients who did not retrieve their result were tested HIV positive. Among them, as shown in Table 2, the female sex (63.5%) was the dominating one. Women not collecting their results were more likely to be tested HIV positive: OR = 1.62, 95% CI: 1.08–2.42; p < 0.05.

**Table 2:** Results of HIV-testing according to the sex

	HIV-test result		Total
	Negative	Positive	
Female	100 (51.8)	129 (63.5)	229 (57.8)
Male	93 (48.2)	74 (36.5)	167 (42.2)
Total	193 (48.7)	203 (51.3)	396 (100)

The most HIV-infected group was the one coming for a voluntary screening (36.5%) followed by associated-diseased patients (33.5%) (Table 1). According to Table 3, young adults (21–35 years) who did not retrieve their HIV result were the most infected by the virus (50.8%).

**Table 3:** HIV-serology in different age groups

Age group (years)	Serology number (%)	
	Negative	Positive
15–20	27 (14.0)	9 (4.4)
21–25	61 (31.6)	18 (8.9)
26–30	42 (21.8)	41 (20.2)
31–35	20 (10.4)	44 (21.7)
36–40	10 (5.2)	42 (20.7)
41–45	16 (8.3)	20 (9.9)
46–50	6 (3.1)	16 (7.9)
51–55	7 (3.6)	8 (4.0)
56–60	1 (0.5)	2 (1.0)
61–65	2 (1.0)	1 (0.5)
66–70	0 (0.0)	2 (1.0)
>70	1 (0.5)	0 (0.0)

## Discussion

This study showed that the percentage of failure to return to receive HIV-test results in the Cameroonian context is almost 10%, which is less than what has been found so far in other studies: 11–48% [9–11]. Though this frequency can be seen as encouraging, many more strategies need to be implemented to reduce this rate, especially targeting young adults (20–35 years) and women, remembering that being acquainted of one’s HIV status for a radical change in HIV-risky behaviours leads to the reduction in the disease spreading [3]. On the other hand, the delay in delivering patients’ results should be shortened.

This low level of failure to retrieve HIV-test results could be explained by the strategy used for pre-test counselling. In fact, a collective followed by an individual counselling was conducted prior to the HIV screening. Nonetheless, we do believe that by reducing the time limit of three days for patients to come back and collect their HIV-test result, this percentage we have obtained could be decreased accordingly. Wiley et al. bolstered for instance that the utilisation of rapid tests for HIV screening along with an immediate delivering of the result have significantly reduced the percentage of failure to return to receive HIV-test results [11]. Furthermore, in order to bring this percentage down to nil, some authors have advocated phone calls as an excellent means to communicate HIV-test results to patients [7, 12], even though this solution may not be without any consequence.

Results from the 2011 Cameroon Demographic and Health Survey revealed a 4.1% HIV seroprevalence [13], more than 10 times lower than what we observed from the entire population who visited the HIV clinic (43.8%), and even lower with regard to patients who did not come back to retrieve their result (51.3%). This high seroprevalence we have witnessed could be justified by the fear of a positive HIV-test subsequent to a risky sexual life [14]. What’s more, our study site is specialised in the management of the HIV/AIDS pandemic; as such, it receives many patients referred from other health facilities to have their HIV status confirmed prior to their follow-up.

In line with other studies, we observed that women were more likely not to retrieve their HIV-test result [9, 11, 15]. Likewise, age was another potentially associated factor of not returning to collect HIV-test results as depicted by other authors [10, 12]. We found indeed that young adults aged 21–35 years had an increased risk to be absent at the appointment to collect their HIV-test result. According to the literature, people with HIV-risky behaviours have an increased likelihood not to come back and collect their HIV test result. For example, youths who have been transfused did not come back to collect their result as they are aware of the risk of HIV transmission through transfusion of contaminated blood [16]. May be our youths did not return to collect their results because they are conscious of their HIV-risky behaviours, especially risky sexual behaviours that could thereby result in an increased HIV seroprevalence among this population. Noubiap et al. showed indeed a high rate of HIV-risky sexual behaviours among youths in Cameroon [17]. We observed that 41 of the 83 youths aged 26–30 years who did not come back to retrieve their HIV-test result (49.4%) had been tested HIV-positive. Patients who visited the clinic for a voluntary HIV screening exhibited a higher rate of not returning to receive their HIV test results. This could perhaps be explained by anguish and the fear of stigmatisation if the result appeared positive [5, 6].

Unfortunately, the cross-sectional design of this study precluded us from investigating all the factors leading to failure to return to retrieve HIV test results, and their magnitude accordingly. Another flaw of this study resides in the fact that we could not seek for patients who did not come to collect their results and simply ask them what have been their motivations, given that our study was retrospective and HIV screening was anonymous as well. Nonetheless, we used strong statistical methods to investigate the risk factors of not returning to collect HIV test results. Another strength of this study is the high study population.

## Conclusions

We found that almost 10% of patients screened for HIV infection did not return to collect their result. The major potential factors associated with the likelihood of not returning to collect HIV-test results included the female sex, young adults and voluntary screening. Many more strategies should be implemented to reduce this percentage, especially targeting young adults (20–35 years) and women, remembering that being acquainted of one’s HIV status for a radical change in HIV-risky behaviours leads to a significant reduction in the disease spreading. The delay in delivering patients’ results should be shortened. Moreover, measures should be taken to increase the level of voluntary HIV-testing.

## Abbreviations

AIDS: acquired immunodeficiency syndrome
CI: confidence interval
HAART: highly active antiretroviral therapy
HIV: Human Immunodeficiency Virus
SD: standard deviation
STROBE: Strengthening the Reporting of Observational Studies in Epidemiology
WHO: World Health Organization
